# Hepatic Steatosis Index in Acromegaly: Correlation with Insulin Resistance Regardless of the Disease Control

**DOI:** 10.1155/2018/5421961

**Published:** 2018-12-19

**Authors:** Alessandro Ciresi, Valentina Guarnotta, Daniela Campo, Carla Giordano

**Affiliations:** Section of Endocrinology, Biomedical Department of Internal and Specialist Medicine (DIBIMIS), University of Palermo, Piazza delle Cliniche 2, 90127 Palermo, Italy

## Abstract

**Objective:**

In acromegaly, both lipotoxicity secondary to GH excess and insulin resistance have a significant impact on the liver. Ultrasonography has shown poor sensitivity in detecting hepatic steatosis and noninvasive methods have been proposed. We evaluated the hepatic steatosis index (HSI), a validated surrogate index of hepatic steatosis, and we correlated it with disease activity and insulin resistance.

**Design:**

Thirty-one patients with newly diagnosed acromegaly were studied at diagnosis and after 12 months of treatment with somatostatin receptor ligands.

**Methods:**

Glucose and insulin levels, surrogate estimates of insulin sensitivity, and hepatic steatosis through ultrasonography and HSI were evaluated.

**Results:**

At diagnosis, ultrasonography documented steatosis in 19 patients (61.2%) while 26 (83.8%) showed high HSI. After 12 months, both GH (*p* = 0.033) and IGF-1 (*p* < 0.001) significantly decreased and, overall, 58% of patients were classified as controlled. Ultrasonography documented steatosis in all the same initial 19 patients, while only 14 patients (45.1%) showed high HSI (*p* < 0.001). A significant reduction in HOMA-IR (*p* = 0.002) and HSI (*p* < 0.001) and increased ISI Matsuda (*p* < 0.001), was documented. The change of HSI from baseline to 12 months was found to be directly correlated with the change of ISI (Rho -0.611; *p* = 0.004) while no correlation was found with the change of GH or IGF-1 levels and other parameters.

**Conclusions:**

In acromegaly, HSI is mainly related with insulin resistance and the reduction of GH and IGF-1 levels, and above all the improvement in insulin sensitivity leads to an improvement of this surrogate index of hepatic steatosis.

## 1. Introduction

Nonalcoholic fatty liver disease (NAFLD) is the most common liver disorder in developed countries, characterized by elevated serum levels of free fatty acids and fatty infiltration of the liver. NAFLD consists of a wide spectrum of conditions, ranging from simple steatosis to nonalcoholic steatohepatitis (NASH), and diagnosis of NASH is based on the histologic examination of liver biopsy specimens [1].

NAFLD, which represents a further expression of metabolic syndrome being strictly linked to obesity, diabetes mellitus, and insulin resistance (IR), has been found to increase the risk for cardiovascular disease, and IR was proposed as a pathogenic factor of NASH through an accumulation of fat within a hepatocyte [2, 3]. In addition, it is well-known that the liver is one of the important target tissues of growth hormone (GH) [4].

GH has physiological effects on glucose metabolism by inducing gluconeogenesis and glycogenolysis and promoting insulin resistance both in the liver and the periphery. In addition, the well-known lipolytic effects of GH stimulate release of free fatty acids (FFA) from the adipose tissue leading to glucose-fatty acid substrate competition and decreased glucose utilisation [5, 6].

Given the main role played by GH in glucose and lipid metabolism, several lines of evidence suggest that GH status is implicated in the development of intrahepatic lipid accumulation. Indeed, both GH excess and deficiency are associated with metabolic disturbances and lipid accumulation in the liver. Low GH levels are associated with hepatic steatosis in patients with NAFLD [7], and GH-deficient adults have a higher incidence of NAFLD [8]. Therefore, NAFLD has emerged as an important form of comorbidity in GH deficiency and it seems to reverse after GH replacement therapy [9].

Conversely, in acromegaly GH excess has a significant impact on adipose tissue and a detrimental effect on glucose metabolism and insulin signaling both at the hepatic and extrahepatic levels. The increased lipolysis in acromegaly results in high circulating FFA levels that may alter adipose tissue deposition, leading to the development of IR [10]. Therefore, in active acromegaly, while body fat depots are diminished, IR located at both hepatic and extrahepatic levels is increased [11].

However, in acromegaly increased lipolysis and IR theoretically have opposite effects on the NAFLD. Indeed, if the lipolytic action of GH may lead to a reduction in visceral and subcutaneous adipose tissue in patients with active disease [12, 13], on the other hand it could be hypothesized that fat deposition in the liver and muscle may represent one of the mechanisms involved in the pathogenesis of IR [14–16], which is impacted by the GH status [17], although there is also evidence against intrahepatic lipid accumulation as one of the underlying mechanisms [18–20].

To date, ultrasonography has shown poor sensitivity in detecting hepatic steatosis and cannot accurately quantify the amount of hepatic fat present [3].

The hepatic steatosis index (HSI) is a simple and efficient screening tool for NAFLD that may be utilized for selecting individuals for liver ultrasonography [21]. HSI discriminates well between patients with and without hepatic steatosis [22]. In addition, HSI has been found to show a significant correlation with fatty liver grade measured by ultrasonography and this finding suggests that HSI reflects not only the presence of NAFLD but also its degree [21]. Therefore, HSI has been proposed as a diagnostic tool for predicting the presence of NAFLD with reliable accuracy and an overall good diagnostic performance [23].

In light of this, we aimed to evaluate the HSI in a group of naive acromegalic patients and to correlate it with disease activity and IR.

## 2. Subjects and Methods

From a total of 75 patients affected by acromegaly who referred at the Endocrinology Section of the University of Palermo from January 2009 to December 2017, we enrolled 31 consecutive patients (17 males and 14 females; aged 51 ± 12 yr, range 32-77 yr) with active newly diagnosed acromegaly and who refused first-line pituitary surgery.

Twenty-eight patients previously treated with surgery or already treated with medical therapy for acromegaly, as well as 4 patients with mixed secreting adenoma or with deficiency of one or more anterior pituitary hormones, were excluded from this study to avoid any impact on the metabolic parameters evaluated. In addition, we excluded 11 patients (in addition to 23 already excluded due to previous surgery) with a previous diagnosis of diabetes mellitus for the same reasons mentioned above and also because they could not undergo oral glucose tolerance test (OGTT) and 1 patient (in addition to 14 already excluded) treated with lipid-lowering drugs. All patients affected by impaired fasting glucose (IFG) or impaired glucose tolerance (IGT) were treated with diet alone. No patients with previous diagnosis of IFG or IGT recruited in the study were already receiving pharmacological treatment.

Disease activity was confirmed by elevated age- and gender-corrected plasma IGF-1 levels and nonsuppressible GH and after OGTT, while the biochemical control of acromegaly during treatment was defined by normal age-adjusted IGF-1 levels and random GH levels < 1 *μ*g/L [24, 25].

In all patients, the MRI scan revealed the presence of a pituitary tumor. The mean duration of the disease was established by patient interview, patient clinical pictures, and onset of osteoarticular symptoms.

All patients underwent first-line medical treatment with depot somatostatin receptor ligands (SRL). Specifically, 19 (61%) received octreotide long-acting release (LAR) (20-40 mg every 28 days) and 12 (39%) lanreotide autogel (ATG) (60-120 mg every 28 days). Octreotide was started at a dose of 20 mg/28 days and lanreotide at a dose of 60 mg/28 days, and dosages were titrated on the basis of GH and IGF-1 at month 3 or 6. After 12 months, in the octreotide group, 10 patients practiced the monthly dose of 20 mg, 5 patients 30 mg, and 4 patients 40 mg; in the lanreotide-group, 5 patients had ATG 60 mg, 5 the monthly dose of 90 mg, and 2 patients 120 mg.

At the time of hospitalization, all patients signed a consent form for the scientific use of their data after a full explanation of the purpose of the study. This study was approved by the Institutional Review Board of the Faculty of Medicine, University of Palermo.

### 2.1. Study Design

Body mass index (BMI), waist circumference (WC), systolic blood pressure (SBP), and diastolic blood pressure (DBP) were measured in all patients. WC was measured at the midpoint between the lower rib and the iliac crest. A 12-hour overnight fasting blood sample was drawn to measure lipid profile (total, high-density lipoprotein (HDL), and low-density lipoprotein (LDL) cholesterol and triglycerides (TG)), hemoglobin A1c (HbA1c), alanine (ALT) and aspartate (AST) aminotransferase, *γ*-glutamyltransferase (GGT), alkaline phosphatase (ALP), total bilirubin, and IGF-1 levels. To normalize IGF-1 for age in individual patients, we calculated the ratio between the IGF-1 level and the upper limit of the normal (ULN) range for age (normal ≤ 1) and the data are presented as IGF-1 ULN. OGTT was performed by measuring plasma blood glucose, insulin, and GH levels every 30 min for 2 h after a 75 g oral glucose load. As surrogate estimates of basal insulin sensitivity, we calculated the homeostasis model assessment of IR (HOMA-IR) index [26], while stimulated insulin sensitivity was measured using the insulin sensitivity index (ISI), a composite index derived from the OGTT and validated by Matsuda and DeFronzo [27].

According to the Adult Treatment Panel III criteria, patients were classified as having abdominal obesity (defined as WC > 102 cm in males and >88 cm in females), reduced HDL (defined as HDL levels < 1.04 mmol/L in males and <1.30 mmol/L in females), raised TG (defined as TG levels ≥ 1.7 mmol/L), elevated blood pressure (defined as ≥130/≥85 mmHg), and raised plasma glucose (defined as fasting glucose ≥ 6.1 mmol/L) [28].

Steatosis was diagnosed by an abdominal ultrasound, routinely performed in all acromegalic patients to evaluate visceromegaly. Ultrasound assessment was performed in fasting subjects on the day of enrollment in the study always by the same radiologist trained for ultrasound techniques and particularly dedicated to liver examination who was unaware of patients' history and aim of the current study. A real-time Hitachi H21 apparatus with a 2 to 5 MHz, convex, multifrequency probe was used.

In addition, we calculated the HSI with the following formula:

(1) $$\begin{array}{c} hepatic\;steatosis\;index\left(HSI\right)=8\times \left(ALT:AST\;ratio\right)+BMI\left(+2,\text{if }female\right), \end{array}$$

and patients were considered to have steatosis if HSI > 36 [21].

### 2.2. Hormone and Biochemical Assays

All biochemical data were collected after overnight fasting. Fasting glucose, ALT, AST, GGT, ALP, bilirubin, and lipid levels were measured in our centralized accredited laboratory with standard methods. Serum insulin was measured by electrochemiluminescence (ECLIA, Elecsys Insulin, Roche, Milan, Italy). The sensitivity of the method was 0.4 *μ*U/mL. The normal range (*μ*U/mL) was 2.6-24.9. Serum GH levels were measured by immunoassay in electrochemiluminescence (ECLIA, Elecsys hGH, Roche, Milan, Italy). The lower limit of detection of the assay was 0.030 *μ*g/L. The intra- and interassay coefficients of variation (CV) were 0.6-5.0 and 3.8-5.0%, respectively. We reported GH concentrations in *μ*g/L of IS 98/574. Serum IGF-I levels were measured by means of a chemiluminescent immunometric assay (Immulite 2000; Diagnostic Products Corp., Los Angeles, CA) using murine monoclonal anti-IGF-I antibodies. The standards were calibrated against the World Health Organization second IS 87/518. The sensitivity was 1.9 *μ*g/L. The intra- and interassay CVs were 2.3-3.9% and 3.7-8.1%, respectively.

### 2.3. Statistical Analysis

The Statistical Packages for Social Sciences SPSS version 19 was used for data analysis. Baseline characteristics were presented as mean ± SD for continuous variables; rates and proportions were calculated for categorical data. The normality of distribution of the quantitative variables was assessed by means of the Kolmogorov-Smirnov test. The differences between paired continuous variables (before and after 12 months of treatment) were analyzed using the Wilcoxon paired test. Simple univariate correlations among continuous variables were determined by Spearman's test. A *p* value of 0.05 was considered statistically significant.

## 3. Results

The clinical and biochemical features of patients are shown in Table 1.

At diagnosis, mean fasting GH values were 11 ± 11.6 *μ*g/L while mean IGF-1 ULN was 2.2 ± 0.9. The mean estimated duration of disease was 5 ± 4.9 years. In the whole cohort of patients, 18 (58%) had IFG or IGT, 15 (48.3%) had hypertension, 9 (29%) had dyslipidemia, and 20 (64.5%) showed increased WC [26]. Two patients (6.4%) reported to moderately consume alcohol, while 7 patients (22.5%) reported being smokers.

Transaminases, GGT, ALP, and bilirubin were within the normal range in all patients. Abdominal ultrasound documented steatosis in 19 patients (61.2%) and biliary lithiasis in 3 patients (9.6%), while 26 patients (83.8%) showed HSI > 36 (Table 1).

After 12 months of treatment, both GH (2.5 ± 1.8 vs. 11 ± 11.6 *μ*g/L; *p* = 0.033) and IGF-1 ULN (1 ± 0.5 vs. 2.2 ± 0.9; *p* < 0.001) significantly decreased. Overall, 18/31 patients (58%) were classified as controlled and 13/31 (42%) as uncontrolled [29].

No significant difference in the prevalence of IFG/IGT, hypertension, dyslipidemia, and increased WC was found from baseline to 12 months. Specifically, at 12 months 16 patients (51.6%) had IFG or IGT, 14 (45.1%) had hypertension, 8 (25.8%) had dyslipidemia, and 22 (70.9%) showed increased WC. The 2 patients (6.4%) mentioned above continued to report moderately consuming alcohol and the 7 patients (22.5%) mentioned continued to report being smokers after 12 months of treatment.

Abdominal ultrasound documented steatosis in all the same initial 19 patients, while biliary lithiasis was found in 6 patients (19.3%). Fourteen patients (45.1%) showed HSI > 36 (vs. 26 patients, 83.8% at baseline; *p* < 0.001). After 12 months, a significant reduction in fasting insulin (8.9 ± 4.9 vs. 16.9 ± 13.9 IU/ml; *p* = 0.014), HOMA-IR (2.4 ± 1.6 vs. 4.3 ± 3.1; *p* = 0.002), and HSI (35.2 ± 4.3 vs. 41.6 ± 5; *p* < 0.001) was documented, with a concomitant significant increase in ISI (5.8 ± 3.3 vs. 3 ± 1.6; *p* < 0.001), while no significant change in other parameters evaluated was found (Table 1).

**Figure 1:**
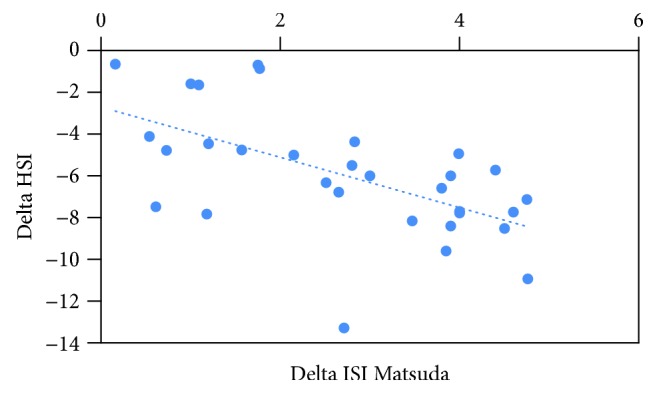
Correlation between the change (delta) of the hepatic steatosis index (HSI) from baseline to 12 months of treatment and delta of ISI Matsuda. Ultrasonography detected hepatic steatosis in 19 (61.2%) patients both at baseline and after 12 months of treatment, while high HSI (defined as >36) was found in 26 (83.8%) patients at baseline and 14 (45.1%) patients after treatment.

In the whole cohort of patients, the change (delta) of HSI from baseline to 12 months was found to be directly correlated with the delta of ISI (Rho -0.611; *p* = 0.004) (Figure 1), while no correlation was found with the delta of GH or IGF-1 levels and other metabolic parameters (Table 2).

When we analyzed patients separately according to disease control, we found no significant difference between controlled and uncontrolled patients in HSI values at both baseline (42.5 ± 5.3 vs. 40.3 ± 4.5; *p* = 0.297) and 12 months of treatment (34.9 ± 5 vs. 35.8 ± 3.4; *p* = 0.613). No significant difference in the number of subject with HSI > 36 (9/18 controlled patients, 50% vs. 5/13 uncontrolled patients, 38.4%; *p* = 0.473) was found between the 2 groups at 12 months of treatment. As in the whole population, both groups of patients showed a significant correlation between delta HSI and delta ISI (*p* = 0.026 and *p* = 0.029 in controlled and uncontrolled patients, respectively).

## 4. Discussion

In this study, we showed that active acromegaly is associated with high HSI, which significantly decreases after treatment with SRL in concomitance with an improvement in insulin sensitivity and regardless of disease control.

It is well-known that active acromegaly is strongly associated with a condition of lipotoxicity and visceral adiposity dysfunction and that IR is one of the main metabolic alterations that characterize it [12, 29, 30].

Both lipotoxicity and IR have a significant impact on the liver. Indeed, in active acromegaly the lipolytic effect of GH excess leads to adipose tissue redistribution [12, 13]. The increased lipolysis secondary to GH excess may lead to a reduction in visceral and subcutaneous adipose tissue in active acromegalic patients [12, 13, 31–33]. However, while body fat depots are diminished, both hepatic and extra-hepatic IR is increased [11]. Indeed, steatosis is a common metabolic feature of acromegaly and fat deposition in the liver and muscle may represent one of the mechanisms involved in the pathogenesis of IR in acromegaly [12, 14–16]although there is also evidence against hepatic lipid accumulation in active acromegaly [20, 34]. Studies have shown that GH signaling limits hepatic lipid accumulation in mice [18, 19] and liver lipid content proved significantly lower in acromegalic patients versus healthy subjects [20].

To date, reliable and accurate data on the prevalence of hepatic steatosis in acromegaly are not available. Hepatic steatosis is commonly detected by imaging such as ultrasound or computed tomography. However, these examinations have poor sensitivity, detect fat only when 20% to 33% of the liver parenchyma is involved, and cannot accurately quantify the amount of hepatic fat present [3].

In the current study, ultrasound showed that about 61% of patients showed steatosis and the same unchanged percentage was documented after 12 months of treatment, although all indexes of liver function were always in the normal range and remained unchanged from baseline to 12 months of treatment. Indeed, ultrasonography is an imperfect gold standard for diagnosis of fatty liver due to imperfect sensitivity and, ultrasound-documented steatosis being a dichotomous variable, any slight changes are difficult to detect and a diagnostic challenge is to accurately detect and quantify the degree of steatosis. For these reasons, noninvasive methods for detecting hepatic steatosis have been proposed [3]. HSI is a simple and efficient screening tool, validated from a large population-based cross-sectional study involving more than 10,700 patients, using BMI and the results of routinely performed laboratory tests to detect NAFLD [21]. HSI has been shown to significantly correlate with ultrasonography fatty liver grade and, consequently, to reflect degree of hepatic steatosis [21, 23].

In this study, we calculated the HSI as a numerical variable which may be considered a reliable surrogate index of steatosis. Twelve months of treatment with SRL was able to significantly reduce HSI in our cohort of patients, and, when we considered the validated cutoff of ≥36 to detect steatosis, we found a significantly reduced percentage of patients with high HSI after SRL treatment than baseline.

Our clinical data are in agreement with those of Madsen et al., who showed no changes in liver enzymes from baseline to 12 months of treatment in acromegalic patients, while a decrease in intrahepatic lipid accumulation was documented by magnetic resonance spectroscopy, confirming the beneficial effect of SRL treatment [34].

In our study, the change in HSI was accompanied by a significant reduction of fasting insulin and improvement in insulin sensitivity and these data are widely documented in previous studies [35]. Indeed, we found a significant decrease in HOMA-IR and an increase in ISI, while no significant change in lipid profile and a slight, although not significant, increase in fasting glucose were documented.

It is indeed well-known that SRL treatment acts centrally not only by inhibiting GH release and consecutively decreasing IR but also by decreasing pancreatic insulin secretion and thus potentially impairing glycemic control [36, 37], although some studies failed to show any change in glucose and insulin metabolism during acromegaly treatment [38].

This suggests that the balance between the effects mediated by the decrease in GH levels and the effects on pancreatic *β*-cells determines whether treatment of acromegaly worsens glucose metabolism or not. Although in this study control of acromegaly was reached by about 58% of patients, quite in line with data in the literature [39–41], we found a significant improvement in both steatosis and insulin sensitivity, regardless of hormonal levels.

Indeed, the significant reduction of GH and IGF-1 levels documented after 12 months of treatment, although they did not reach the recommended cutoff for cure of acromegaly [25], was associated with significant metabolic changes, improvement in insulin sensitivity, and reduction of HSI.

It is well-known that treatment for acromegaly promotes changes in body composition and, although no significant change in body weight was documented in our study, a significant redistribution of fat mass is hypothesized, with a decrease in fat mass in visceral organs [1, 13, 33, 42, 43]. However, we found a higher number of subjects, although not significant, with increased WC after treatment compared to baseline. This result can be explained by the reduction of excessive lipolytic activity secondary to excess GH after 12 months of medical treatment.

Supporting the link between the HSI and insulin sensitivity, the univariate analysis confirmed a significant negative correlation between delta of HSI and delta of ISI Matsuda, while no correlations were found with GH or IGF-1 levels.

In our opinion, the direct correlation between delta HSI and delta ISI confirms the hypothesis that insulin resistance represents a pathogenic factor of steatosis and, specifically, that in acromegaly the metabolic improvement during treatment has a greater weight than the biochemical one.

The lack of correlation between delta HSI and delta HOMA-IR is probably due to the inability of HOMA-IR to reliably assess insulin sensitivity. Indeed, the HOMA-IR just represents a basal index of insulin resistance closely related to basal glucose and insulin levels, while ISI Matsuda, which derives from glucose and insulin levels during OGTT, shows a different behavior and certainly represents a more reliable index of insulin sensitivity than the basal HOMA-IR.

A weakness of our study could be the limited number of patients enrolled, as we enrolled only those who refused surgery as first-line treatment. In addition, in our cohort we did not have overt diabetic patients, to avoid interference with metabolic evaluation. Another limitation of the study is probably the lack of direct hepatic data based on histologic examination of liver biopsy specimens, to date considered the gold standard for diagnosing NAFLD, because its widespread use is limited by the risk associated with an invasive procedure, cost, and sampling error [1, 44].

In conclusion, in acromegaly HSI is mainly related with IR and the reduction of GH and IGF-1 levels, and above all the improvement in insulin sensitivity leads to an improvement of this surrogate index of hepatic steatosis, which should always be evaluated in the follow-up of acromegalic patients.

## Figures and Tables

**Table 1:** Clinical and biochemical features of patients at diagnosis and after 12 months of treatment.

	Baseline	After 12 months	*p*
	Subjects (%)	Subjects (%)	
Gender			
Males	17 (55)	—	—
Females	14 (45)		
	Mean ± SD	Mean ± SD	
Age (years)	51.3 ± 12.4	—	—
Duration of disease (years)	5 ± 4.9	—	
Fasting GH (*μ*g/L)	11 ± 11.1	2.5 ± 1.8	0.033
IGF-1 ULN	2.2 ± 0.9	1 ± 0.5	<0.001
BMI (kg/m^2^)	29.9 ± 3.8	29.9 ± 3.9	0.974
WC (cm)	102 ± 11	102 ± 13	0.885
Systolic blood pressure (mmHg)	129 ± 25	122 ± 14	0.330
Diastolic blood pressure (mmHg)	77 ± 9	73 ± 9	0.224
Fasting glucose (mmol/L)	5.76 ± 1.78	5.89 ± 1.15	0.646
Fasting insulin (IU/mL)	16.9 ± 13.9	8.9 ± 4.9	0.014
HOMA-IR	4.3 ± 3.1	2.4 ± 1.6	0.002
ISI Matsuda	3 ± 1.6	5.8 ± 3.3	<0.001
Total cholesterol (mmol/L)	5.1 ± 0.8	4.6 ± 1.3	0.133
HDL cholesterol (mmol/L)	1.2 ± 0.2	1.1 ± 0.3	0.269
LDL cholesterol (mmol/L)	3.1 ± 0.8	2.8 ± 1	0.326
Triglycerides (mmol/L)	1.6 ± 1.1	1.2 ± 0.6	0.146
AST (IU/L)	16 ± 6	17 ± 6	0.970
ALT (IU/L)	16 ± 7	15 ± 4	0.473
GGT (IU/L)	16 ± 11	24 ± 14	0.371
ALP (IU/L)	69 ± 16	73 ± 10	0.425
Total bilirubin (*μ*mol/L)	12.9 ± 2.5	12.6 ± 8.9	0.964
HSI	41.6 ± 5	35.2 ± 4.3	<0.001
Hepatic steatosis (ultrasound)	19 (61.2)	19 (61.2)	—
HSI > 36	26 (83.8)	14 (45.1)	<0.001

IFG: impaired fasting glucose; IGT: impaired glucose tolerance; WC: waist circumference; AST: aspartate transaminase; ALT: alanine transaminase; GGT: *γ*-Glutamyltransferase; ALP: alkaline phosphatase; HSI: hepatic steatosis index.

**Table 2:** Correlation between the change (delta) of the hepatic steatosis index (HSI) and change of metabolic and hormonal parameters from baseline to 12 months of treatment (univariate analysis).

	Delta HSI	
	Rho	*p*
Delta GH (*μ*g/L)	0.143	0.736
Delta IGF-1 (*μ*g/L)	0.230	0.279
Delta fasting glucose (mmol/L)	0.058	0.789
Delta fasting insulin (IU/mL)	0.113	0.667
Delta HOMA-IR	0.009	0.970
Delta ISI Matsuda	-0.611	0.004

## Data Availability

The data used to support the findings of this study are available from the corresponding author upon request.
